# First-in-Human Experience With the ModulHeart Device for Mechanical Circulatory Support and Renal Perfusion

**DOI:** 10.1016/j.jscai.2022.100449

**Published:** 2022-09-17

**Authors:** Gabriel Georges, François Trudeau, Jade Doucet-Martineau, Maxime Rochon, Jeannot Potvin, Adrian Ebner, Philippe Généreux

**Affiliations:** a Quebec Heart and Lung Institute, Quebec, Quebec, Canada; b Puzzle Medical Devices Inc, Montreal, Quebec, Canada; c Centre Hospitalier de l’Université de Montréal, Montreal, Quebec, Canada; d Sanatorio Italiano, Asuncion, Paraguay; e Gagnon Cardiovascular Institute, Morristown Medical Center, Morristown, New Jersey

**Keywords:** first-in-human, heart failure, left ventricular assist device, mechanical circulatory support, percutaneous coronary intervention

## Abstract

**Background:**

ModulHeart (Puzzle Medical Devices Inc) is a modular device providing hemodynamic support through 3 endovascular pumps inserted in series and assembled in parallel into a self-expandable anchor implanted in the descending aorta. The current study evaluates the feasibility and safety of cardiorenal support with ModulHeart among patients undergoing high-risk percutaneous coronary intervention (PCI).

**Methods:**

This study was a prospective, single-center, first-in-human study. The primary end point was procedural success, defined as successful delivery, function, and removal of the ModulHeart device. Secondary end points included pump hemodynamics, cardiac hemodynamics, and urine output.

**Results:**

On June 28 and 29, 2022, 4 patients were enrolled and underwent high-risk PCI with ModulHeart implanted via transfemoral approach. All 4 patients achieved procedural success. The mean delivery time was 8 minutes, the mean support time was 49 minutes, and the mean pump removal time was 7 minutes. The mean pressure gradient across the pump was 5 ± 2 mm Hg. Under ModulHeart support, cardiac index increased by 25%, central venous pressure decreased by 37%, and left ventricular end-diastolic pressure decreased by 78%. Urine output increased by ∼9-fold after 15 minutes of support. No device malfunction or procedural or device-related adverse events occurred. There was no evidence of pump thrombosis. All 4 patients were alive at 30 days.

**Conclusions:**

This first-in-human study demonstrated the feasibility and safety of cardiorenal support with ModulHeart among patients undergoing high-risk PCI. ModulHeart demonstrated significant improvement in cardiac output, left ventricular end-diastolic pressure, and urine output. Future studies are planned to assess outcomes associated with ModulHeart support in patients with heart failure.

## Highlights

• ModulHeart is a novel percutaneous mechanical circulatory support device implanted in the abdominal aorta
• ModulHeart’s 3 pumps are inserted in series and assembled in parallel in an anchor
• The ModulHeart first-in-human study in 4 patients showed 100% procedural success
• Cardiac output increased by 25%, urine output increased by 9-fold, and left ventricular end-diastolic pressure decreased by 78%
• No hemolysis or no pump thrombosis occurred

## Central Illustration

**Figure:**
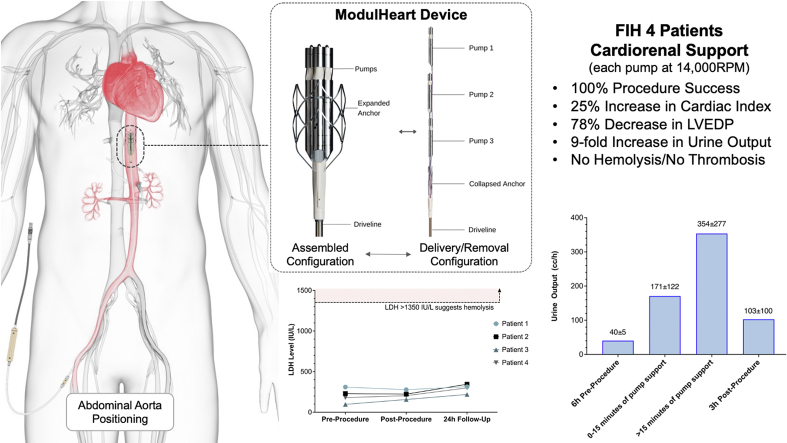

## Introduction

Percutaneous hemodynamic support options for patients with heart failure or those undergoing high-risk percutaneous coronary intervention (PCI) are limited. Currently approved devices have suffered from intrinsic limitations, such as lack of stability, hemolysis, bleeding, and stroke, which are mainly caused by their intracardiac/transvalvular position and high pump rotational speed.^1^, ^2^, ^3^, ^4^ A potential increase in mortality, morbidity, and costs with the use of these devices in different clinical settings, such as acute myocardial infarction and cardiogenic shock, has raised some safety concerns regarding their use and points toward the need to develop novel, safe, and effective mechanical support devices.^5^^,^^6^

**Figure 1:**
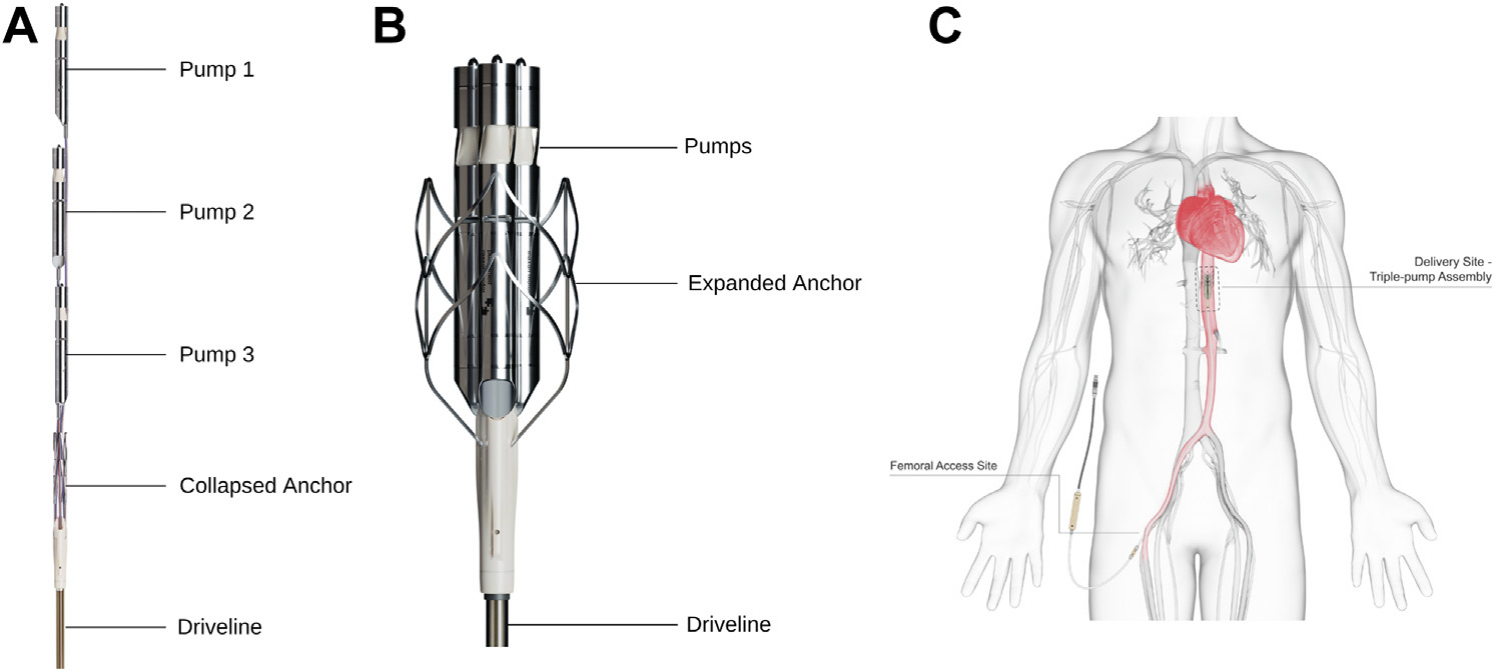
**The ModulHeart device.** (**A**) Components of the ModulHeart device before insertion and assembly: 3 pumps in series, a self-expandable nitinol anchor (collapsed), and its driveline to power the device. (**B**) The ModulHeart device in its assembled configuration, with the 3 pumps anchored in parallel within a self-expandable anchor (expanded). (**C**) Positioning of the ModulHeart within the descending abdominal aorta.

The ModulHeart device (Puzzle Medical Devices Inc) is a novel modular pump implanted percutaneously, providing hemodynamic support through 3 endovascular pumps inserted in series (Figure 1A) and assembled in parallel into a dedicated self-expandable anchor (Figure 1B) in the descending aorta (Figure 1C). The presence of multiple (3) pumps assembled in parallel allows for a cumulatively higher flow than that achieved with a single pump, with each pump rotating at a lower speed, resulting in a lack of blood element damage. Indeed, a high rotational speed has been associated with blood damage, such as von Willebrand factor (vWF) destruction and hemolysis, impacting the efficacy and safety of those devices and precluding longer-term support.^4^ The current study was designed to evaluate the feasibility and safety of ModulHeart for mechanical circulatory support (MCS) and renal perfusion (cardiorenal support) among patients undergoing high-risk PCI.

## Methods

This first-in-human (FIH) study is a single-arm, open-label, single-center prospective evaluation of cardiorenal support in patients undergoing complex or high-risk PCI. The study was performed at the Sanatorio Italiano, Asuncion, Paraguay. The study was approved by the Paraguay National Board of Health Bioethics Committee. Each patient provided informed consent.

### Study population

Enrolled patients required nonurgent complex or high-risk PCI, defined as at least 1 of the following: (1) unprotected left main PCI, (2) multivessel (>1 vessel) PCI, (3) bifurcation lesions PCI, (4) severely calcified lesion PCI, (5) last patent conduit to the heart PCI, (6) saphenous graft or arterial bypass PCI, or (7) presence of left ventricular dysfunction (left ventricular ejection fraction, 20%-50%). The exclusion criteria included cardiogenic shock, anatomical characteristics that would preclude safe placement of a 22F sheath, and severe calcification of the descending aorta. Consented subjects underwent screening procedures and were scheduled for their procedure.

### Procedure

**Figure 2:**
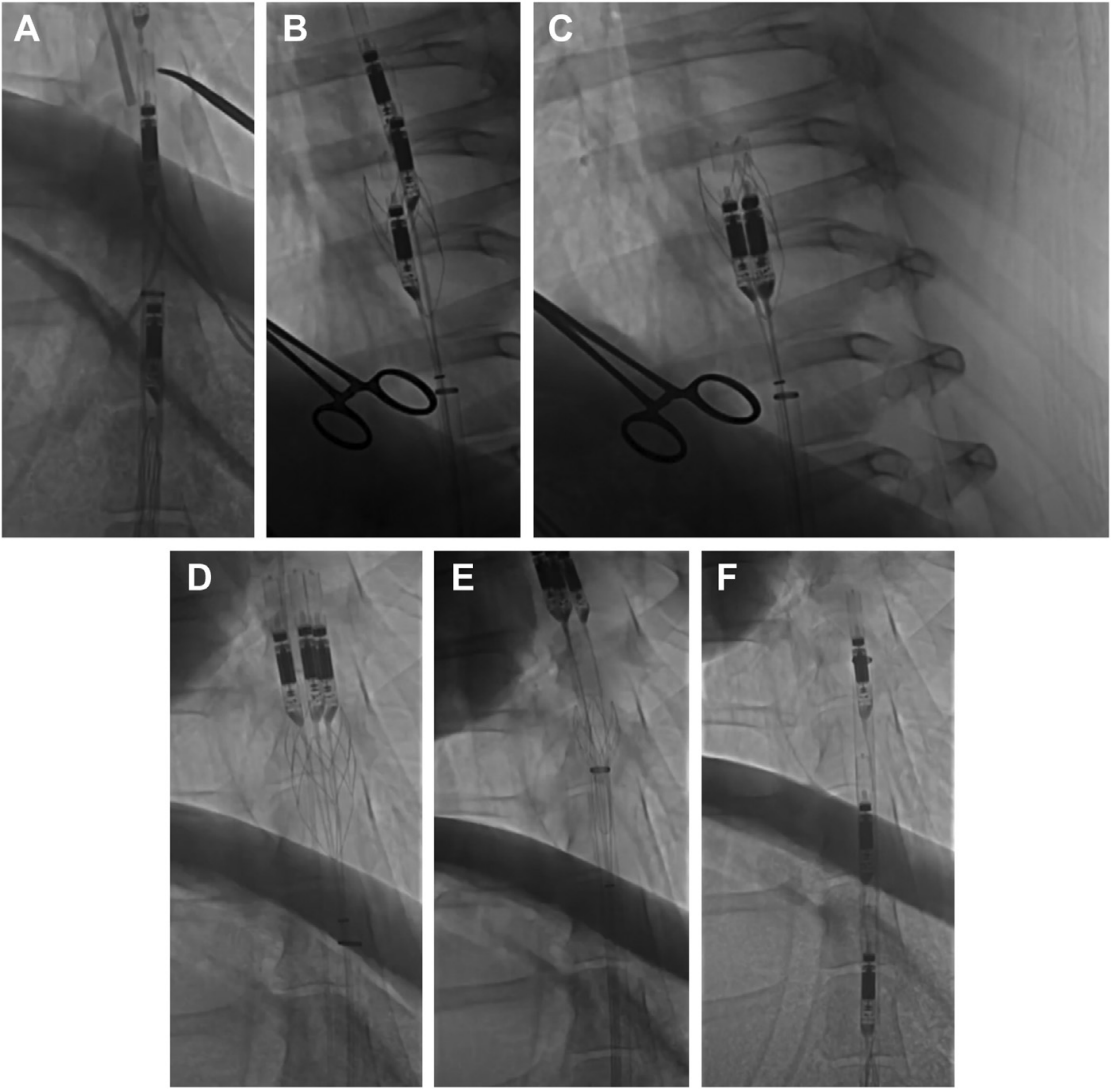
**The ModulHeart device implantation and retrieval steps.** (**A**) ModulHeart device in the delivery configuration, with multiple pumps aligned in series within an endovascular sheath. (**B**) Assembly of the ModulHeart device. Each individual pump is positioned into an anchor from an in-series configuration to a parallel configuration. (**C**) The ModulHeart device in its final assembled configuration, with the 3 pumps assembled in parallel within the self-expandable anchor. (**D**) The pumps are disassembled from the anchor. (**E**) The anchor is recollapsed for device removal. (**F**) The 3 ModulHeart pumps are resheathed for final removal (Supplemental Video 1).

The ModulHeart device was implanted percutaneously via the right femoral artery using the standard percutaneous technique. Preclose using 2 Perclose ProGlide (Abbott) was performed. PCI was performed via the right radial artery using a 6F guide catheter. Right heart catheterization was performed via the left femoral vein using a 6F Swan-Ganz catheter. Cardiac output was derived using the Fick method. The left radial artery was used to deliver a 6F pigtail catheter positioned in the descending aorta (thoracic) to measure inflow pump pressure; the left femoral artery was used to deliver a 6F pigtail catheter positioned in the abdominal aorta to measure outflow pump pressure. Patients were anticoagulated with unfractionated heparin to achieve an activated clotting time of >250 seconds and received dual antiplatelet therapy. The ModulHeart device was inserted through a 22F sheath before the beginning of the PCI and removed after the completion of the PCI. The device was delivered in the proximal abdominal aorta above the level of the renal arteries at the level of the T11 vertebral body. Figure 2 and Supplemental Video 1 show the implantation and retrieval steps of the ModulHeart device. Hemodynamic parameters were measured at baseline (before pump initiation), during the procedure (with each pump unit of the ModulHeart device set at a constant rotational speed of 14,000 revolutions per min [RPM], with an estimated flow rate of 4 L/min), and after the pump was removed. These included left ventricular end-diastolic pressure (LVEDP), central venous pressure (CVP), pulmonary artery pressure, arterial pressures at the inlet and outlet of the pump, and cardiac output. Values obtained under ModulHeart support were compared with the values obtained with no support, defined as the mean of 15 minutes before ModulHeart initiation and 15 minutes after ModulHeart removal. Blood samples were obtained at baseline (before the procedure), at the end of the procedure, and 24 hours after the procedure to assess for hemolysis via measurement of lactate dehydrogenase (LDH) and plasma-free hemoglobin. Urine output was monitored with a bladder Foley catheter.

### Study end points

The primary end point was procedural success, defined as successful delivery, function, and removal of the ModulHeart device. Safety end points included hemolysis assessment, freedom from pump displacement, and procedural device-related major cardiovascular adverse events, defined as death, stroke, or evidence of pump thrombosis, including embolic events. Secondary end points included pump hemodynamics (pressure gradient across the device), cardiac hemodynamics (cardiac output, CVP, pulmonary artery pressure, and LVEDP), and periprocedural renal parameters (urine output [3 hours before the procedure, during the procedure, and 6 hours after the procedure] and creatinine level [before the procedure and 24 hours after the procedure]). Procedural and device-related adverse events were recorded until discharge. Patients were followed for 30 days. Outcomes were reported by the principal investigator (A.E.) at the study site.

### Statistical analysis

Results were reported using descriptive statistics. The *t* test was used to compare recorded variables at different time points. All measures were tested at a significance level of .05. Data were analyzed using Prism (GraphPad Software).

## Results

### Patient characteristics

On June 28 and 29, 2022, 4 patients met the inclusion/exclusion criteria and were enrolled in the current study. Baseline characteristics and high-risk PCI criteria are presented in Table 1. All patients were men, with a mean age of 64 ± 2 years. The mean left ventricular ejection fraction was 51 ± 5%, and the mean creatinine level was 1.17 ± 0.24 mg/dL. All patients had complex coronary disease requiring revascularization.

**Table 1:** Baseline characteristics.

	Patient 1	Patient 2	Patient 3	Patient 4
Age, y	66	64	61	66
Sex	Male	Male	Male	Male
BMI, kg/m^2^	30	34	25	30
LVEF, %	50	55	55	45
Creatinine, mg/dL	1.25	0.89	1.07	1.45
Coronary anatomy	Multivessel disease with bifurcation lesions	Multivessel disease, RCA CTO	Proximal LAD CTO	Complex proximal LAD bifurcation perfusing large territory
Right CFA mean diameter, mm	8.5	9.0	8.3	9.0

BMI, body mass index; CFA, common femoral artery; CTO, chronic total occlusion; LAD, left anterior descending coronary artery; LVEF, left ventricular ejection fraction; RCA, right coronary artery.

### Procedure and clinical outcomes

The study device was delivered percutaneously via the right common femoral artery, with a mean delivery time of 8 ± 1 minutes, a mean support time of 49 ± 8 minutes, and a mean removal time of 7 ± 1 minutes. The current consumption of the pump was stable during the entire procedure. The anchor and the 3 docked pumps showed great stability, with no device movement during the procedure. The study device was successfully deassembled and removed with no complications. Hemostasis was achieved percutaneously in all cases (2 Perclose ProGlide), which was helped with a crossover balloon for optimization. All 4 patients underwent successful implant and removal of the study device, with great function and no evidence of procedure- or device-related complications. No pump thrombosis occurred. Postprocedure aortogram and femoral angiography demonstrated no aorta or iliofemoral vascular complications. No clinically significant bleeding occurred, and no transfusions were required. At the 30-day received in-person follow-up care, all 4 patients were alive, with no strokes or other adverse cardiovascular events.

### Hemodynamic parameters

During ModulHeart support, the systolic and diastolic arterial pressure gradients increased by 6 ± 3 mm Hg (*P* < .0001) and 4 ± 2 mm Hg (*P* < .0001), respectively, across the device, with a mean arterial pressure gradient increase of 5 ± 2 mm Hg (*P* < .0001) (Table 2). The CVP decreased by 37% (10 mm Hg vs 6 mm Hg; *P* = .05) and LVEDP decreased by 78% (9 mm Hg vs 2 mm Hg; *P* = .006) during pump support compared with no pump support. Cardiac index increased by ∼25% during ModulHeart support compared with no support (2.4 ± 0.3 vs 3.1 ± 1; *P* = .47).

**Table 2:** Hemodynamic impact of ModulHeart support

	No support	ModulHeart support	*P* value
Proximal systolic pressure, mm Hg	109 ± 25	110 ± 24	.93
Distal systolic pressure, mm Hg	109 ± 25	116 ± 24	.47
Proximal diastolic pressure, mm Hg	52 ± 18	54 ± 13	.81
Distal diastolic pressure, mm Hg	52 ± 18	58 ± 12	.40
Pump pressure gradient			
Systolic, mm Hg	0	6 ± 3	<.0001
Diastolic, mm Hg	0	4 ± 2	<.0001
Mean, mm Hg	0	5 ± 2	<.0001
PAP systolic, mm Hg	48 ± 10	42 ± 11	.24
PAP diastolic, mm Hg	12 ± 6	7 ± 5	.05
PAP mean, mm Hg	24 ± 5	19 ± 5	.03
Central venous pressure, mm Hg	10 ± 4	6 ± 3	.05
LVEDP, mm Hg	9 ± 5	2 ± 1	.006
Cardiac index, L/min/m_2_	2.4 ± 0.3	3.1 ± 1	.47

Values are mean ± SD.

LVEDP, left ventricle end-diastolic pressure; PAP, pulmonary artery pressure.

### Renal function

**Figure 3:**
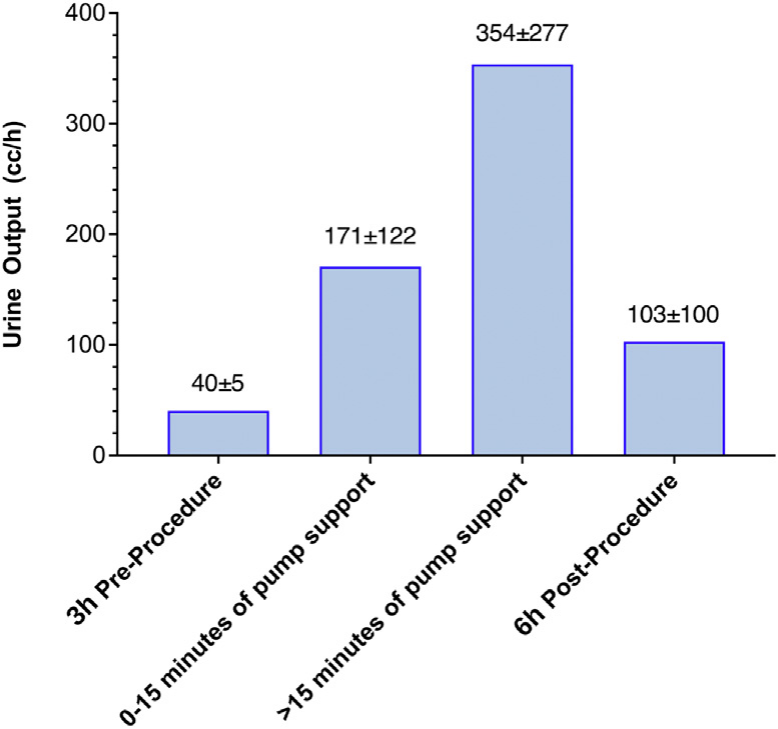
**Urine output before and after ModulHeart device support.** Urine output increased by ∼4-fold in the first 15 minutes of support, followed by a ∼9-fold increase after 15 minutes of support compared with baseline. Urine output remained elevated 6 hours after procedure compared with baseline.

The impact of ModulHeart support on urine output is shown in Figure 3. The mean urine output at baseline 3 hours before the procedure was 40 ± 5 cc/h. It increased by ∼4-fold (171 ± 122 cc/h) in the first 15 minutes of pump support and by ∼9-fold after 15 minutes of support (354 ± 277 cc/h) compared with baseline. Urine output remained elevated after the procedure, with a mean of 103 ± 100 cc/h. There was no significant change in the creatinine level (0.70 ± 0.28 mg/dL vs 0.76 ± 0.23 mg/dL; *P* = .34) or the estimated glomerular filtration rate (129 ± 75 mg/dL vs 113 ± 55 mg/dL; *P* = .26) before the procedure and 24 hours after the procedure.

### Hemolysis assessment

**Figure 4:**
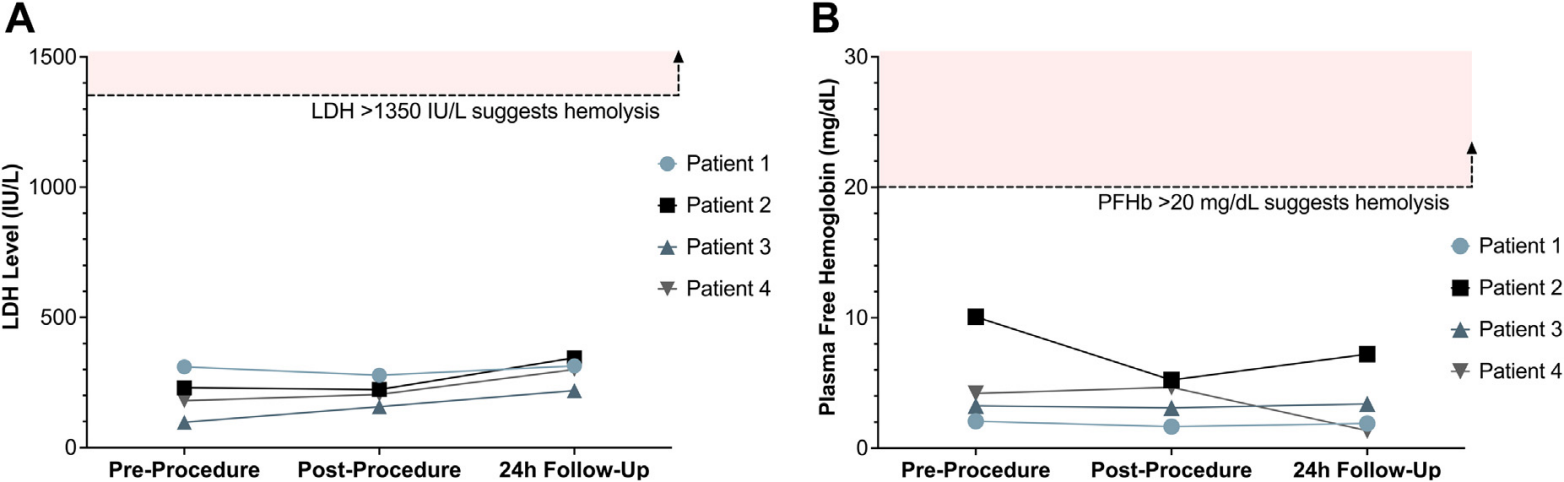
**Lactate dehydrogenase (LDH) and plasma-free hemoglobin (PFHb) before and after ModulHeart device.** (**A**) No changes in LDH or (**B**) PFHb were observed during or after ModulHeart device support, suggesting lack of hemolysis.

No change in the LDH level was observed during or after ModulHeart support. LDH levels remained lesser than the hemolysis threshold of 3 times the upper limit of the normal LDH range (before the procedure, 204 ± 89 IU/L; after the procedure, 216 ± 50 IU/L; and at the 24-hour follow-up, 295 ± 54 IU/L) (Figure 4A). Similarly, no change in plasma-free hemoglobin was observed, with values 5 times lesser than the hemolysis threshold of 20 mg/dL (before the procedure, 4.90 ± 3.56 mg/dL; after the procedure, 3.66 ± 1.61 mg/dL; and at the 24-hour follow-up, 3.46 ± 2.65 mg/dL) (Figure 4B).

## Discussion

The aim of this study was to demonstrate the safety and feasibility of cardiorenal support during high-risk PCI using the ModulHeart device implanted in the descending aorta. The main findings of the current study are as follows: (1) a novel MCS device was developed to improve hemodynamics and renal perfusion, (2) the FIH experience demonstrated procedural and device success without hemolysis, (3) ModulHeart demonstrated local and systemic hemodynamic benefits, and (4) ModulHeart demonstrated improvement in renal parameters.

### Unique characteristics of ModulHeart

ModulHeart is a novel and unique modular pump assembled inside the patient. The device’s design allows for hemodynamic support through multiple (3) parallel endovascular pumps. Compared with conventional single transcatheter pumps, ModulHeart can generate more flow (up to 10 L/min) at lower speeds, leading to a decreased risk of hemolysis, decreased shear stress, and decreased blood element damage, such as destruction of vWF, known to be associated with an increased bleeding risk. In addition, by being implanted within a self-expandable anchor in the descending aorta, ModulHeart could nullify the risks of strokes compared with transvalvular devices and could be used in patients with severely calcified/stenotic aortic valves, mechanical aortic valves, or left ventricle thrombus or patients with significant aortic insufficiency. Additionally, the large cell-sized self-expandable nitinol anchor ensures pump stability, allowing for continuous and uninterrupted support and patient ambulation. Previous animal models have demonstrated the stability of the anchor and pumps in an ambulatory animal (data on file; Puzzle Medical Devices Inc). This may provide a significant advantage over transvalvular MCS devices, especially if the duration of support is prolonged and if offered to ambulatory patients (out of hospital). ModulHeart is positioned in the descending aorta (distal abdominal aorta) at the level of T11, which has been demonstrated to be the most optimal position for an intra-aortic entrainment pump. Indeed, intra-abdominal positioning (T10-T12), compared with thoracic positioning (T5-T6), was shown to provide a higher reduction in left ventricle afterload and higher increase in cardiac output.^7^ Importantly, no significant changes in carotid or coronary pressures were noted in this position, similar to our preclinical animal work (data on file; Puzzle Medical Devices Inc). Table 3 summarizes key features of currently available or under-investigation devices for cardiorenal support.

**Table 3:** Comparison of approved mechanical circulatory support devices and mechanical circulatory support under investigation with publicly available data

	IABP	Impella CP (Abiomed)	Impella 5.5 (Abiomed)	Peripheral VA ECMO	Aortix (Procyrion)^a^	ModulHeart (Puzzle Medical Devices)^a^
Device description	Counter-pulsation balloon	1 single microaxial pump	1 single microaxial pump	Extracorporeal centrifugal pumps with oxygenator membrane	1 single microaxial pump	3 individual microaxial pumps assembled in parallel in a self-expandable anchor
Position	Thoracic aorta	Transvalvular	Transvalvular	Iliofemoral artery and vein	Abdominal aorta	Abdominal aorta
Access	8F catheter, femoral artery	14F catheter, femoral artery	23F catheter, axillary artery	∼15-24F catheter, femoral artery; ∼19-25F catheter, femoral vein	18F catheter, femoral artery	22F catheter, femoral artery, 16F catheter next generation
Working RPM	N/A	23,000 RPM for 0.9 L/min, up to 46,000 RPM for 3.7 L/min	17,000 RPM for up to 1.9 L/min; 33,000 RPM for up to 5.5 L/min	∼4,000 RPM for ∼4 L/min	25,000 RPM for ∼3.5 L/min	14,000 RPM for 4 L/min
Maximum flow rate	N/A	Up to 3.7 L/min at 46,000 RPM	Up to 5.5 L/min at 33,000 RPM	Up to ∼8 L/min at ∼5,000 RPM	Up to 5 L/min at 30,000 RPM	Up to 10 L/min at 25,000 RPM
Indications	High-risk PCI, cardiogenic shock	High-risk PCI, cardiogenic shock	Cardiogenic shock	Cardiogenic shock	High-risk PCI, ADHF	High-risk PCI, ADHF, CHF
Pros	Small insertion profile; low cost; increased coronary flow	Direct LV unloading; approximately small insertion profile	Direct LV unloading	Concomitant gas exchange; high flow rate	Increased renal flow; conceptually no stroke risk	Increased renal flow; self-expandable nitinol anchor; low blood damage; conceptually no stroke risk; high flow rate
Cons	Indirect LV unloading; poor degree of unloading	Bleeding; pump stability issues; limited flow rate; hemolysis and vWF degradation; risk of stroke; contraindicated/not ideal in patients with AS, AI, or mechanical AVR; current device not suitable for outpatient use	Large insertion profile; surgical access; pump stability issues; hemolysis and vWF degradation; risk of stroke; contraindicated/not ideal in patients with AS, AI, or mechanical AVR; current device not suitable for outpatient use	Retrograde flow; no LV unloading; large insertion profile; bleeding; limb ischemia	Indirect LV unloading; large insertion profile; risk of blood damage because of a high rotational speed	Indirect LV unloading; large insertion profile in current version (upcoming version <16F and supporting axillary access)

ADHF, acute decompensated heart failure; AI, aortic insufficiency; AS, aortic stenosis; AVR, aortic valve replacement; CHF, chronic heart failure; VA-ECMO, veno-arterial extracorporeal membrane oxygenation; IABP, intra-aortic balloon pump; LV, left ventricle; N/A, not applicable; PCI, percutaneous coronary intervention; RPM, revolutions per minute; vWF, von Willebrand factor.

a Investigational devices.

Recently, a small series of 6 patients undergoing high-risk PCI with hemodynamic support with an 18F single microaxial pump device (Aortix; Procyrion, Inc) located in the abdominal aorta was reported.^8^ Similar to our findings, urine output increased significantly with support, with favorable impact on LVEDP and cardiac output, supporting the concept of kidney perfusion via intra-aortic fluid entrainment. That being said, potential limitations of the Aortix device include the use of a single pump with higher rotational speed (22,000-37,000 RPM),^7^ increasing the risk of blood element damage, hemolysis, and subsequent bleeding, especially if longer-term support is contemplated. Each pump unit of the ModulHeart runs at 14,000 RPM, which we believe is the optimal speed, balancing flow and blood element preservation (vWF/hemolysis). Additionally, ModulHeart uses a unique anchoring mechanism (self-expanding nitinol stent) to ensure pump stability and minimize aortic wall trauma during insertion and retrieval.

### Procedural and device success

The current FIH study demonstrated the safety and feasibility of cardiorenal support using the ModulHeart device in the context of high-risk PCI. The endovascular assembly and deassembly of the device (pumps and anchor) and the complete removal of the device was done safely and efficiently (∼7 minutes), with no vascular or bleeding issues. Similar to prior animal work, no hemolysis was noted during or after the procedure, and no pump thrombosis occurred. Those findings are encouraging, and future device iterations, with lower insertion profile, will ensure to preserve the safety of this procedure and allow for longer duration of support.

### Hemodynamic effects

During ModulHeart support, we observed an increase in cardiac index and reduction in LVEDP and CVP compared with baseline. Those results suggest that although ModulHeart does not directly unload the left ventricle, the device does improve cardiac function. Potential mechanisms include the generation of a negative pressure head at the inlet of the pump, which may reduce left ventricular afterload, and the creation of a Venturi effect by the high-pressure flow exiting at the outlet of the pump. Increased diuresis may have also contributed to the improvement of hemodynamic parameters by optimizing volemia and left ventricle filling pressure.

### Renal effects

During pump support, we demonstrated a mean pressure gradient across the pump of ∼5 mm Hg at a pump speed of 14,000 RPM, with dramatic improvement in urine output. Our preclinical in vivo animal studies showed that the pressure gradient across the pump gradually increased with increasing pump speeds, with up to 13 mm Hg of mean arterial pressure gradient at 22,000 RPM (data on file, Puzzle Medical Devices Inc). These results suggest that intra-aortic fluid entrainment increases renal blood flow and perfusion, leading to increased diuresis. Therefore, it is expected that in patients with acute or chronic decompensated heart failure refractory to optimal medical therapy, the ModulHeart device may help to relieve congestion. Of note, diuresis remained more than ∼2.5-fold of the baseline values 6 hours after the procedure. Although further data are necessary to confirm this finding, increasing renal perfusion with the ModulHeart device may provide benefits beyond the implant period and potentially increase medical therapy responsiveness.

### Future directions

The current FIH study was performed among a population of patients undergoing high-risk PCI with some degree of left ventricular dysfunction. The next studies will target patients with acute and chronic decompensated heart failure with more profound reduced ejection fraction and requiring prolonged hemodynamic support. Whether randomized trials comparing optimal medical therapy with MCS for patients presenting with decompensated heart failure would be needed remains to be determined. Importantly, formal assessment of cerebral perfusion (steal phenomenon) would be important to demonstrate the safety of prolonged intra-abdominal support.

### Study limitations

The current study represents the FIH evaluation of the new ModulHeart device to support cardiorenal function. Several limitations of the present study should be acknowledged. First, the duration of ModulHeart support was ∼1 hour. A longer duration might have been more informative regarding the beneficial impact on cardiac and kidney function. Second, although no vascular or bleeding complications occurred in this study, the current ModulHeart device requires 22F catheter access. Future iterations of the device will have a ≤16F catheter insertion profile with a residual drive line footprint of ≤10F and be suitable for axillary access. Third, no vWF measurements were performed before or after ModulHeart implantation in the current study. vWF is known to be a more sensitive measure of shear stress and blood element damage compared with LDH or plasma-free hemoglobin (hemolysis).^4^ That being said, a prior animal study demonstrated preservation of vWF during 6 hours of ModulHeart support at different pump speeds, which compared favorably to other MCS devices, such as Impella CP (Abiomed) or Impella 5.0 (Abiomed), which destroyed the vWF within 30 minutes after device activation because of a higher rotational speed of their single pump (data on file; Puzzle Medical Devices Inc).^4^

## Conclusion

This FIH study demonstrated the feasibility and safety of cardiorenal support with the ModulHeart device among patients undergoing high-risk PCI. ModulHeart demonstrated significant improvement in cardiac output, LVEDP, and urine output. Given these results, further investigation using the ModulHeart device for longer periods of time are underway, such as in patients with acute or chronic decompensated heart failure refractory to optimal medical therapy (Central Illustration).

**Central Illustration:**
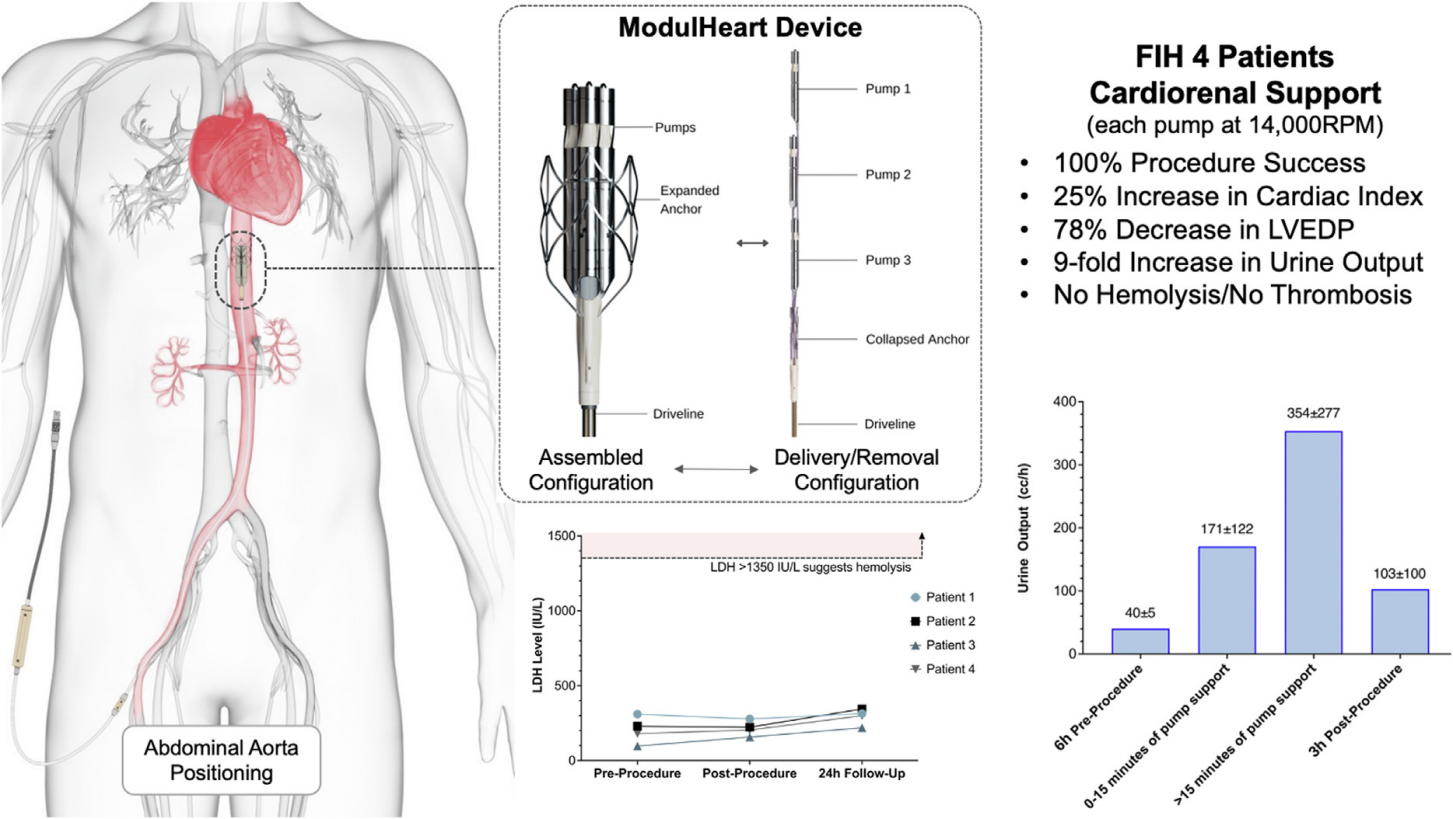
The ModulHeart device and its components: 3 pumps inserted in series that are assembled in parallel in its final configuration, a self-expandable nitinol anchor, and its driveline attachment. ModulHeart is positioned within the abdominal aorta, approximately at the level of T11. This first-in-human (FIH) study among 4 patients undergoing high-risk percutaneous coronary intervention with cardiorenal support using the novel ModulHeart device demonstrated 100% procedural success and favorable hemodynamic impacts with 25% increase in cardiac index, 78% decrease in left ventricular end-diastolic pressure (LVEDP), 9-fold increase in urine output after 15 minutes of support, and no hemolysis or thrombosis. LDH, lactate dehydrogenase; RPM, revolutions per minute.

## Supplementary material

Supplemental Video 1 The ModulHeart device (Puzzle Medical Devices Inc) is inserted percutaneously via transfemoral artery and consists of 3 endovascular pumps initially inserted in series and then assembled in parallel into a self-expandable nitinol anchor, positioned in the abdominal aorta. The ModulHeart is removed percutaneously, with the 3 endovascular pumps being disassembled from the anchor, followed by the anchor being collapsed and resheathed with the 3 endovascular pumps in series.
